# Avoiding a Med-Wreck: a structured medication reconciliation framework and standardized auditing tool utilized to optimize patient safety and reallocate hospital resources

**DOI:** 10.1186/s40545-021-00296-w

**Published:** 2021-01-19

**Authors:** Ali Elbeddini, Sarah Almasalkhi, Thulasika Prabaharan, Cindy Tran, Mohamed Gazarin, Ahmed Elshahawi

**Affiliations:** 1 Winchester District Memorial Hospital, 566 Louise Street, Winchester, ON KK0C2K0 Canada; 2 Leslie Dan Faculty of Pharmacy, University of Toronto, 144 College St, Toronto, M5S 3M2 Canada; 3 University of Toronto, Medical Sciences Building, Room 3157, 1 King’s College Circle, Toronto, ON M5S 1A8 Canada

**Keywords:** Medication reconciliation, Auditing tool, Medication errors, COVID-19, Cost-effective analysis

## Abstract

**Background:**

The incidence of preventable adverse drug events (ADE) is approximately one medication error per patient per hospital-day. A quality medication reconciliation (MedRec) process is a crucial intervention used to reduce ADE in the hospital and community setting. Amid the coronavirus disease 2019 (COVID-19) pandemic, preventing medication errors is vital to avoid patient readmission, reduce disease complications, and reduce cost and patient burden on the healthcare system.

**Objectives:**

To develop a standardized MedRec framework that can be implemented in all healthcare settings to reduce patient and staff harm during COVID-19. Also, to create a standardized auditing tool used to assess the quality of the MedRec process and allow for continuous quality improvement.

**Methods:**

A multi-site gap analysis (MGA) was performed to collect observational data that were collected from four different healthcare sites (two hospitals, a long-term care facility, and a community pharmacy). MGA consists of collecting data across several sites which answer a standardized questionnaire. A standardized MedRec framework and auditing tool were developed based on the gaps observed in each site and literature reviews.

**Results:**

A standardized MedRec process was not implemented in any of the observed sites. The healthcare sites lacked a designated MedRec team and training related to the MedRec process leading to multiple discrepancies at discharge. Patients were not counselled on changes to home medications, and a discharge report was often not provided upon discharge. Communication mechanisms between community pharmacies and hospital physicians are not available or easily accessible.

**Conclusion:**

The proposed structured MedRec framework is vital to reduce medication errors and patient harm amid COVID-19. Moreover, the comprehensive auditing tool developed in this study allows for continuous quality improvement resulting in superior quality care, reduction of workflow inefficiencies, cost savings on hospital readmissions, and overall enhanced healthcare system performance.

## Introduction

Adverse drug events (ADE) are a leading cause of injury and death within health care systems around the world [1–3]. Many of these events occur due to poor communication when care is transferred during hospital admissions, between wards, and on discharge to the community or to a residential care facility. In Canada, approximately 50% of hospital medication errors occur during transitions of care, and roughly 30% of these errors have the potential to cause patient harm [4, 5]. These errors may occur at various stages, such as when obtaining the patient’s best possible medication history (BPMH), when recording the medications in the medical record, and prescribing medications on admission, transferring to another ward, and discharging. In a population of patients discharged from an internal medicine service, 23% of patients experienced an adverse event and 72% of those were medication-related [6]. Furthermore, up to 67% of patients’ BPMH recorded on admission to hospital have one or more discrepancies between medication taken in hospital and medications taken at home [5].

A standardized MedRec process and auditing tool has high potential to reduce patient risk, reduce readmissions, and optimize crucial transitions in the system. Medication discrepancies are both common and costly presenting a significant risk of harm to patients [7–9]. Amid a global pandemic involving COVID-19, standardized hospital processes are crucial to reduce preventable ADEs, ensure patient and staff safety, and ensure continuation of care when patients transition home. The standardized MedRec framework and auditing tool developed in this study can be implemented during this unprecedented time to provide key components to make workflow more time-efficient, reduce medication errors, and improve MedRec quality. All these benefits will reduce harm to patients and staff secondary to unnecessary COVID-19 exposure.

This is an observational study used to collect data on the MedRec process from multiple health care settings. The purpose of this manuscript is to observe the gaps in the MedRec process and to standardize the MedRec process. The primary objective is to implement a structured MedRec framework. The secondary objective is to develop a MedRec auditing tool. This tool can be used to monitor compliance and quality, improve patient care, and can be shared with decision-makers and stakeholders to illustrate the cost-effectiveness of this strategy. Developing a standardized MedRec framework and auditing tool reduces ADE and inappropriate medication selection during COVID-19. Additionally, a standardized MedRec process is hypothesized to reduced hospital costs and reduce the burden on our healthcare system during COVID-19.

## Methods

### MedRec data collection

A MedRec Gap Analysis (MGA) was performed at four sites, Winchester District Memorial Hospital (WDMH), FHT in Ontario, Cornwall Long Term Care (LTC) facility, and a local community pharmacy in order to qualitatively describe the discrepancies at different stages of the MedRec process.

MGA consists of collecting data across several sites which answer a standardized questionnaire. The MedRec process was observed at each site by a registered pharmacist. Data related to discrepancies and procedure details were collected at admission, transition of care, and discharge. The multidisciplinary approach to the MedRec process was assessed by separately observing staff involved in completing a BPMH, writing medication orders, and counselling patients at discharge. The standardized questions used for MedRec data collection at each site are presented in Table 1.

**Table 1:** MedRec gap analysis questionnaire

Questions	Data collection sites			
	WDMH	FHT Ontario	Cornwall LTC	Local community pharmacy
Who conducts the BPMH?	Ward nurses	Pharmacy technician	Pharmacist	Not applicable MedRec list is reviewed by community pharmacist and reconciled based on conversation with clinical pharmacist in the hospital/LTC Other questions are not applicable
Are all staff trained to collect BPMH?	Some	Yes	Yes	
Is there a MedRec process incorporated into workflow?	No	Yes	Yes	
How often are BPMH obtained before medication orders?	Over 80%	90%	75%	
Howe often was the patient and/or caregiver interviewed when collecting the BPMH?	50%	100%	Over 80%	
Do physicians request a BPMH to be completed by pharmacy staff?	No	Yes	Yes	
How is patient information communicated to community pharmacists and family physicians?	Fax	Face-to-face communication between physician and pharmacist	Fax and phone calls	
Are patients counselled on changes to their medications at discharge?	Yes, however only if discrepancies were found	Yes	Yes	

### Standardized MedRec tool development

The development of a standardized MedRec Tool was based on the data collected from MGA. The gaps observed at each site were compared and considered during development (Table 2). Literature reviews, expert knowledge, and clinical practice experiences were used to formulate solutions to the observed discrepancies, which were incorporated into the MedRec tool.

**Table 2:** MedRec Gaps at WDMH, FHT Ontario, Cornwall LTC, and local community pharmacy

Gaps	WDMH	Cornwall LTC	FHT Ontario	Local community pharmacy
#1	Nurses are filling out the MedRec on admissions	Patient’s own document sheet (PODS) not given to all patients	Patients do not receive a complete list of medications from community pharmacy	Lack of communication between hospital physician and community pharmacist
#2	BPMH are collected through single source instead of 2–3 sources to verify the information	PODS not given to community pharmacies	The discharge summary report has discrepancies	Discharge report is missing information like limited use codes
#3	MedRec on transition of care is missed most of the time	No information provided for where community pharmacies can contact to clarify discrepancies—most staff unwilling to help once patient is discharged	Patients/caregivers are not interviewed often when obtaining a BPMH	There are often discrepancies between medications prescribed by hospital physician and family physician
#4	MedRec on discharge do not include patient counseling points. Also, patient medication changes are not always given to patients upon discharge	PODS not reviewed with patients	BPMH is not commonly done proactively	Lack of laboratory results hinders ability to monitor patients on new, changed medications
#5	Communicating the changes are not always shared with community pharmacist, long-term care facilities and family doctors	Discharge prescriptions and PODS/summary of visit are not sent to the patient’s community pharmacy upon discharge		MedRec often not completed accurately. Patient medication list is often missing over-the-counter medications

## Results

### MedRec data collection

The answers to the MGA questionnaire at each site are presented in Table 1. This questionnaire was only applicable to WDMH, FHT in Ontario, Cornwall LTC and several differences in the procedures for MedRec were identified. Different healthcare practitioners were responsible for the conduction the BPMH at the 3 sites. Only WDMH did not have a MedRec process incorporated into workflow. All 3 locations had a high percentage of BPMH conducted before ordering medications with Cornwall LTC with a lowest score of 75%. Fifty or more percentage of caregivers were interviewed at each collection site, with WDMH with the lowest score of 50%. Only the physicians at both FHT Ontario and Cornwall LTC request for BPMH to be conducted by pharmacy staff.

### Standardized MedRec tool

Detailed information related to gaps in the MedRec process at each site is presented in Table 2. Many common gaps in the MedRec process were identified across the different sites. For instance, at WDMH and FHT in Ontario, there was a lack of cross-referencing the BPMH with multiple sources to verify information. Furthermore, patients often did not receive a documented list of their own medications at Cornwall LTC and FHT in Ontario. There was also a lack of consistent communication between the institutions and other sites in the community.

## Discussion

MedRec is a responsibility for everyone in the circle of care. The challenge regarding MedRec is that many institutions fail to perceive that MedRec as part of a broader medication management system with clear accountability, responsibility, and delineated roles. Instead, MedRec is delegated as a checklist or a task that everybody is responsible to complete individually, but often leading to poor-quality results. Additionally, many current processes fail to take into account that certain aspects of MedRec require multiple trained personnel (e.g., pharmacy technicians, student pharmacists) to complete. MedRec should be a process taught explicitly, frequently evaluated and reviewed periodically for quality assurance.

MedRec is the formal process during which health care providers develop a BPMH, which is compared and reconciled with a patient’s active medication orders. As acknowledged by several international patient safety organizations including the World Health Organization, this reconciliation process is completed to avoid medication errors including omissions, duplications, dosing errors, and drug interactions that may subsequently lead to patient harm, particularly at transitions of care. The BPMH is developed by working closely with patients, families, and caregivers to obtain a comprehensive and accurate medication list. While the concept and value of MedRec is relatively straightforward, obtaining an accurate list of all medications that the patient takes at home can be challenging as patients often have multiple comorbidities, medications, and prescribers. Furthermore, there are many stakeholders—community pharmacists, primary care providers, and long-term care facilities involved in a MedRec, each contributing value to the overall process.

**Fig. 1:**
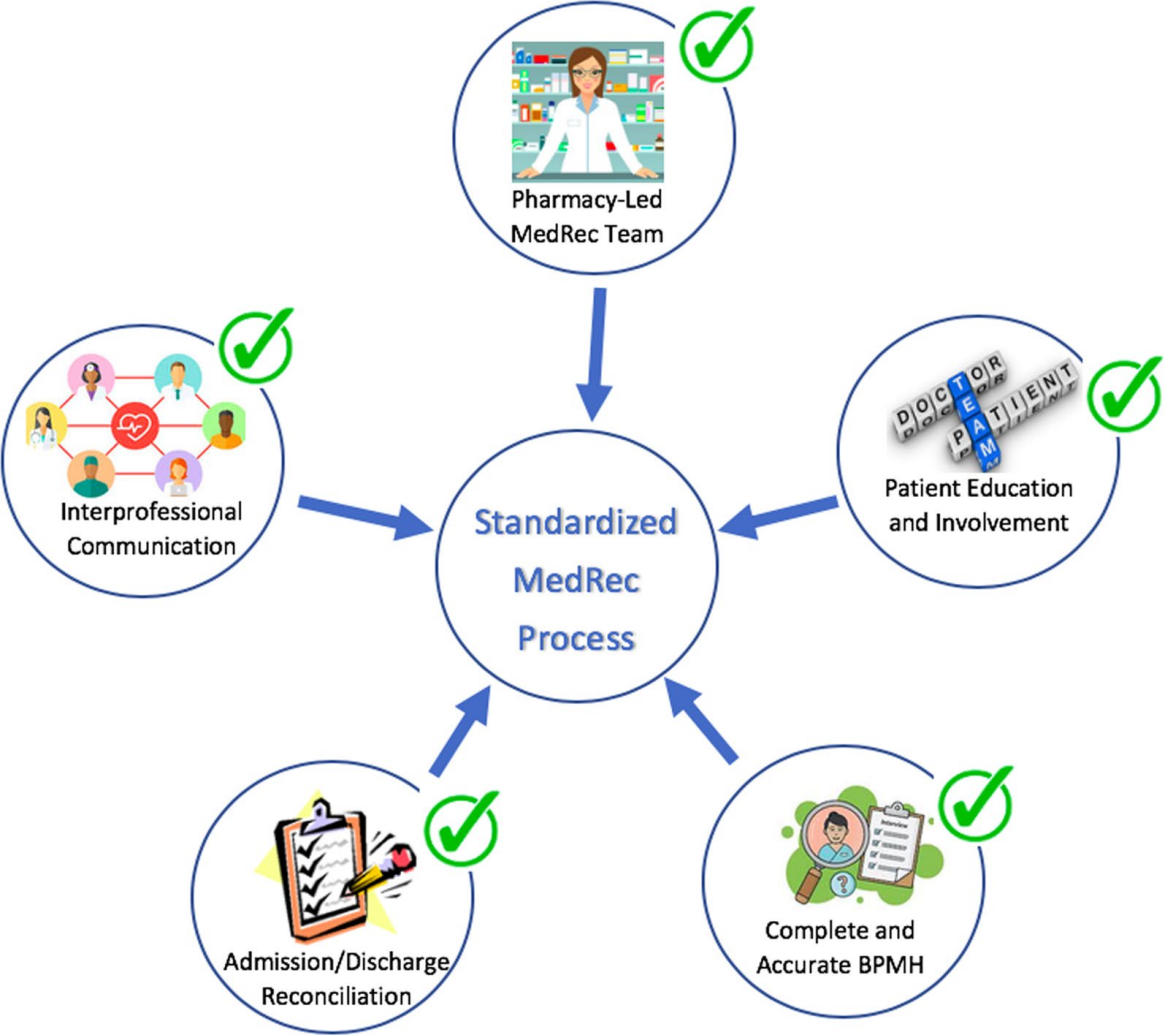
Standardized MedRec components. Five key components were identified for the MedRec process: (1) pharmacy-led MedRec team, (2) patient education and involvement, (3) complete and accurate BPMH, (4) admission/discharge reconciliation, and (5) interprofessional communication

Many medication errors can be prevented through a thorough and accurate MedRec process and by effectively communicating any medication changes to patients and their health care providers at all transitions of care [10, 11]. However, a MedRec is often performed in various ways by different members of the health care team and managed differently at each facility, creating variability in data gathering. A MedRec can also be managed by nurses, pharmacy staff, physicians, or some combination of the aforementioned. This complexity and integration of many moving parts speaks to the need for the establishment of a structured formal process to obtain a complete and accurate medication history by using a best possible medication history (Fig. 1).

### Standardized MedRec framework

The BPMH is the tip of the iceberg. It is a pharmacotherapeutic evaluation completed by a healthcare professional to ensure the therapy is appropriate. Failure to complete this step results in an inappropriate medication list being provided to the next healthcare provider, resulting in a cascade of inappropriate medication management. A quality BPMH is used to rationalize optimal drug therapy for patients by understanding the patients journey with their medications. For example, antimicrobial drug resistance is mitigated when healthcare professionals are aware of the previous antibiotic therapy a patient has taken based on their BPMH. Additionally, medication therapeutic monitoring can be initiated early in patient care when a quality BPMH is obtained from patients. Based on the common gaps identified from MGA, the following standardized MedRec framework was determined:

#### Step 1: formulate a pharmacy-led MedRec team

**Fig. 2:**
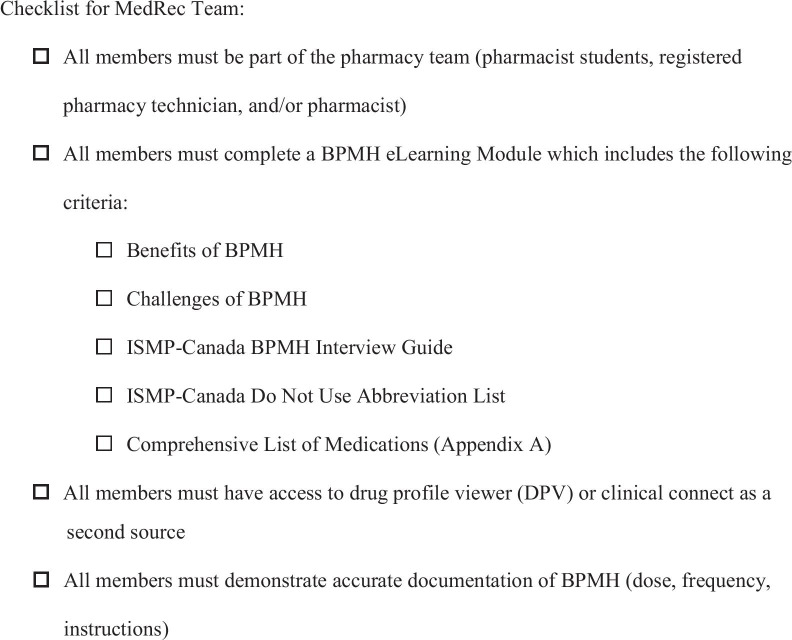
MedRec team requirements

The ideal method to perform an accurate MedRec begins with the establishment of a designated and trained MedRec team of registered pharmacy technicians, pharmacy students, and/or pharmacists who will collect the BPMH from multiple reliable sources. A 2016 systemic review involving 17 studies and 21,342 patients, demonstrated that a pharmacy-led MedRec program compared to usual care reduced the rate of all-cause readmissions by 19%, all-cause ED visits by 28%, and ADE-related hospital readmission by 67% [9]. A checklist, such as in Fig. 2, should be used to ensure an appropriate and trained MedRec team is established and maintained.

#### Step 2: aim to obtain BPMH proactivity

A proactive approach involves obtaining a BPMH before admission orders are written. The MedRec process should ideally be initiated as early as patient arrival at the emergency department (crucial to obtain a clear and concise list of medications on admission, in order to prevent any unintentional discrepancies).

#### Step 3: obtain an accurate BPMH

A successful MedRec starts by obtaining an accurate BPMH:

- Review any pre-established background information on the patient, such as age
- Assess if the patient is willing and able to give reliable history information (some patients may have memory loss or other mental disabilities)
- Identify what language the patient speaks at home to anticipate any communication barriers.

#### Step 4: identify discrepancies between BPMH and medication orders

There are two types of discrepancies that we may find on admission—intentional and unintentional discrepancies. Intentional discrepancies are when the prescriber changes or omits drugs that the patient was regularly taking at home. Undocumented intentional discrepancies are when the prescriber intentionally changes or omits medications and does not document the reason.

Any medication changes upon admission due to the presenting condition must be documented clearly. If admission orders have been already written by admitting physicians, the list could be used to reconcile the medicine's orders on the medications chart on admission. The BPMH should travel with the patient if the patient is transferred from the emergency department to the inpatient ward, from the intensive care unit to ward, from hospital to home, residential care facility, or to other hospitals.

#### Step 5: create a PODS at discharge

Sixty-three percent (63%) of medication incidents take place at discharge [7, 11]. Make sure to give the patient a copy of the most updated list with all the medications. The pharmacist should review the new medication list and make clinical decisions on the appropriateness of each medication. Ensure that patients are aware of any changes. Communicate the changes to family physician and community pharmacy or long-term care facility/nursing homes. Clearly document any changes, so that it is evident they are intentional changes. Follow up with patient to check on quality care during the transition of care. If the patient started the new medications or had medication adjustments, follow up and address any questions.

### Standardized MedRec Auditing Tool

The MedRec Auditing Tool was divided into 2 separate tools. The first auditing tool focuses on the pharmacy-led MedRec team, while the second auditing tool focuses on the MedRec process. Each question in the auditing tool is given a score of 1 or 0 based on corresponding answers of yes or no, respectively. The score for each question is added up to produce and average MedRec score for every patient file being audited. Based on Institute for Safe Medication Practices (ISMP) Canada, a total of 20 patients should be audited each month to ensure high-quality MedRec processes are taken place [8]. To calculate an overall average MedRec score, the total number of patient files who received a perfect score in all categories of the MedRec Auditing Tool is divided by the total number of audited patients. For example, if only 5 out of 20 patients received a perfect score, then the average MedRec Score would be 25% (5/20 = 0.25 × 100 = 25%).

The goal of formulating a standardized auditing tool is to allow for continuous quality improvement (CQI). Systematically monitoring, examining, and evaluating healthcare processes using a standardized auditing tool creates an opportunity to determine the success or failure of current methods. Monthly auditing the MedRec process results in reduction of workflow inefficiencies, improvement of quality care, and enhancement of overall healthcare system performance. To perform CQI, desired outcomes and measurements must be set. The two auditing tools presented in this study provide essential measurement components used to determine the success of a MedRec process in hospital settings. Table 3 illustrates the components for a pharmacy-led MedRec tool. Quarterly monitoring of the pharmacy-led MedRec team using this auditing tool allows for changes to be made on a regular basis to optimize quality care. Furthermore, monthly evaluation of hospital MedRec processes using the standardized MedRec Auditing Tool (Table 4) identifies specific areas requiring further improvement. The auditing tools provided in this study set key components, based on research and expert opinion, to allow healthcare management leaders to continuously evaluate performance and set desired target goals to improve quality care. Future aspirations would be to comprehensively and systematically validate these tools in forthcoming studies. Also, standardized auditing tools can give insight on gaps in the MedRec process associated with the current COVID-19 pandemic, which can be used for future quality improvement initiatives during unprecedented situations.

**Table 3:** Pharmacy-led MedRec Team Auditing tool

Auditing questions	MedRec score (yes = 1; no = 0)
Are all members of MedRec team trained using the MedRec training requirements?	
Was an accurate BPMH obtained on admission?	
Was the BPMH obtained proactively?	
Was the patient and/or caregiver interviewed?	
Were 2 or more sources used to obtain an accurate medication list?	
Is the BPMH documented properly (i.e., accurate dose, frequency, and route)	
MedRec Team Score (out of 6)

**Table 4:** Standardized MedRec Auditing Tool

Auditing questions	MedRec score (yes = 1; no = 0)
Was an accurate BPMH obtained on admission?	
Was an accurate BPMH obtained on internal transfer? (If N/A = score of 1)	
Was an accurate BPMH documented at discharge?	
Were discrepancies between BPMH and medication administration record (MAR) record identified and resolved within 24 h of admission?	
Were discrepancies between BPMH and MAR identified and resolved at discharge?	
Were formulary/non-formulary auto-substitutions in the hospital reverted to what the patient was taking prior hospitalization? (If N/A = score of 1)	
Was a standardized discharge report (i.e., new, continued, stopped, and/or changed medications) faxed to the patient’s primary pharmacy?	
Did the patient receive a PODS at discharge?	
Was the patient counselled by pharmacist on all medications (including monitoring) at discharge?	
Was cost of medications and insurance coverage discussed and addressed with the patient?	
Is there a mechanism of communication between community pharmacist, family physician, and hospital physician?	
MedRec Score (out of 11)	

### Is there a business case related to the MedRec process?

A reflection of the quality of the transitional care and MedRec process is demonstrated through hospital readmissions. Preventing ADE is associated with decreased length of hospital stay, rates of hospital acquired infections and mortality risk, as well as improvement of quality adjusted life years (QALYs) [12–14].

A 2016 discrete-event simulation model, based on published literature data, estimated the total cost of preventable ADEs to be $472 per patient. This model indicated that using a MedRec process as an intervention reduced medication discrepancies by 52%, which translates to a reduced cost of $266 per patient after accounting for intervention costs (i.e., pharmacy technician and pharmacist time and salary). Moreover, the model shows that only a 10% reduction in medication discrepancies will cover the initial cost of MedRec intervention [15]. The incidence of preventable ADE is approximately 1 medication error per patient per hospital-day. If a hospital has 50 beds and each patient has at least 1 medication error (with a cost of $266) prevented by a MedRec pharmacy-led team, this translates to $13,300 per day or $4.85 million annually in cost savings.

According to the Canadian Institute for Health Information, the cost of 30-day readmission for acute care patients is, on average, 42% (or $3117) more than the initial admission cost, resulting in an average cost of $10,404 per hospitalization. Researchers indicated that roughly 9% to 59% of readmissions are preventable, corresponding to $162 million to $1.06 billion, respectively, which can be reallocated to improving other aspects of quality care [16, 17]. Therefore, MedRec is one type of cost-effective strategy used to mitigate the risk and cost associated with hospital medication errors and preventable readmission.

### The MedRec framework amid COVID-19 pandemic

A standardized MedRec framework is vital during COVID-19 pandemic. The results of quality BPMH and MedRec at transition of care minimizes ADE, reduces unnecessary hospital costs, and prevents over burdening the healthcare system during unprecedented times. Appropriate medication management in the hospital and community settings creates a circle of care which is resilient to medication errors and inappropriate medication selection. In order to overcome the uncertainty of COVID-19, the healthcare system is required to formulate a strong interprofessional team with standardized MedRec framework that enables continuity of care and appropriate medication management for all patients—especially COVID-19 positive patients.

The limitations of this study include; (1) the use of only three healthcare sites, (2) the MedRec auditing tool was not validated, and (3) gaps in the MedRec process was not observed on COVID-19 positive patients.

## Conclusion

In conclusion, a standardized MedRec framework has clinical impact on reducing ADE, medication errors, and hospital readmissions. The MedRec framework in this study identified five key components for the MedRec process: (1) a pharmacy-led and trained MedRec team, (2) complete and accurate BPMH, (3) patient education and involvement in changes to medications (4) MedRec at admission, transfer, and discharge, and (5) interprofessional collaboration within the hospital and expanding to community settings. Furthermore, implementing a comprehensive auditing tool allows for continuous quality improvement resulting in superior quality care, reduction of workflow inefficiencies, cost savings on hospital readmissions, and overall enhanced healthcare system performance.

## Data Availability

Data sharing does not apply to this article as no datasets were generated or analyzed during the current study.

## Appendices

### Appendix A: Medication categories for BPMH interview

**Table:**

Medication history checklist	
Prescription medicines Inhalants Nasal sprays Sleeping tablets Oral Contraceptives, hormone replacement therapy Over-the-counter medicines Analgesics Gastrointestinal drugs (for reflux, heartburn, constipation, diarrhea) Cannabis products Nicotine replacement therapy (NRT) and vaping products Shared medication from friends or neighbors	Topical Medicines (e.g. Creams, ointments, lotions, patches) Inserted medicines (nose/ear/eye drops, suppositories) Injectable products Recently completed courses of medicine Social or Recreational drugs Intermittent medicines (e.g. Weekly or twice weekly or on an as-needed basis) Chemotherapy Drugs Sample medications Vitamins and natural supplements Medications provided by specialists e.g. ocular injections Antibiotic use in the past 3 months

### Appendix B: Principles of MedRec Framework

Figure 3.

## Abbreviations

COVID-19: Coronavirus disease 2019
ADE: Adverse drug event
MedRec: Medication reconciliation
BPMH: Best possible medication history
CQI: Continuous quality improvement
PODS: Patient’s own document sheet
MAR: Medication administration record
ISMP: Institute for Safe Medication Practices
QALY: Quality adjusted life years

## References

[1] Preventing medication errors. Institute of Medicine; 2006.

[2] Baker GR, Norton PG (2004). The Canadian Adverse Events Study: the incidence of adverse events among hospitalized patients in Canada. Can Med Assoc J.

[3] Terry A, Mottram C, Round J, Firman E, Step J, Bourne J (2005). A safer place for patients: learning to improve patient safety.

[4] Vira T, Colquhoun M, Etchells EE (2006). Reconciliable differences: correcting medication errors at hospital admission and discharge. Qual Saf Healthcare.

[5] Cornish PL, Knowles SR, Marcheso R, Tam V, Shadowiz S, Juurlink DN, Etchells EE (2005). Unintended medication discrepancies at the time of hospital admission. Arch Intern Med.

[6] Place M (2007). Technical patient safety solutions for medicines reconciliation on admission of adults to hospital.

[7] Forster AJ, Murff HJ, Peterson JF, Gandhi TK, Bates DW (2003). The incidence and severity of adverse events affecting patients after discharge from the hospital. Ann Intern Med.

[8] Medication reconciliation in acute care. Patient Safety Institute; 2017

[9] Mekonnen AB, McLachlan AJ, Jo-anne EB (2016). Effectiveness of pharmacist-led medication reconciliation programmes on clinical outcomes at hospital transitions: a systematic review and meta-analysis. BMJ open.

[10] Hamilton H, Gallagher P, Ryan C, Byrne S, O'Mahony D (2011). Potentially inappropriate medications defined by STOPP criteria and the risk of adverse drug events in older hospitalized patients. Arch Intern Med.

[11] Forster AJ, Murff HJ, Peterson JF, Gandhi TK, Bates DW (2005). Adverse drug events occurring following hospital discharge. J Gen Intern Med.

[12] Buckley MS, Harinstein LM, Clark KB, Smithburger PL, Eckhardt DJ, Alexander E, Devabhakthuni S, Westley CA, David B, Kane-Gill SL (2013). Impact of a clinical pharmacy admission medication reconciliation program on medication errors in “high-risk” patients. Ann Pharmacother.

[13] Scahill S, Harrison J, Carswell P (2009). Organisational culture: an important concept for pharmacy practice research. Pharm World Sci.

[14] Collaboration O, Newton PN, Bond KC, Babar Z (2020). COVID-19 and risks to the supply and quality of tests, drugs, and vaccines. Lancet Global Health.

[15] Najafzadeh M, Schnipper JL, Shrank WH, Kymes S, Brennan TA, Choudhry NK (2016). Economic value of pharmacist-led medication reconciliation for reducing medication errors after hospital discharge. Am J Manag Care.

[16] All-cause readmission to acute care and return to the emergency department. Ottawa: Canadian Institute for Health Information; 2012.

[17] Sullivan C (2005). Medication reconciliation in the acute care setting: opportunity and challenge for nursing. J Nurse Care Qual.

